# Patient-centered high-value integrated care

**DOI:** 10.2478/abm-2022-0025

**Published:** 2023-06-16

**Authors:** Editorial Office of Asian Biomedicine

**Affiliations:** Faculty of Medicine, Chulalongkorn University, Bangkok 10330, Thailand

Patients with chronic physical and mental conditions want to understand how their sickness or treatment will affect their lives, how to maintain self-care away from the clinical setting, and what impacts their illness will have upon their loved ones and finances as well as the extent up to which these impacts are likely in turn to affect their future [1]. There is a great deal of variation in the clinical practices of individual providers, and approaching any of these does not involve a guarantee concerning appropriateness of the cost and desirability of the treatment outcome [2]. Therefore, high-value care affecting the lives and livelihood of patients cannot be based solely on professional judgment. The Choosing Wisely campaign, initiated by the American Board of Internal Medicine Foundation, has developed clinical practice guidelines for clinicians and patients to guide choices of therapeutics, diagnostic tests, procedures, operations, and even decisions regarding whether to admit a patient to the hospital or schedule an outpatient follow-up. This idea is to limit overuse that does not add value for patients and may even cause harm. Available at www.choosingwisely.org, it is a campaign to engage physicians and patients in conversations about unnecessary tests, treatments, and procedures [3]. To be effective, it is suggested that low-value versus high-value care should be discussed during the clinical encounters between patients, their families, and physicians [4]. In the discussions, it is important to identify the barriers to patient involvement and adherence to high-value care, which include discussions about cost, outcomes, complications of interventions, and the implications that proper care, or a dearth of it, can have on their lives and livelihood.

In defining the outcomes, it is important to discuss the clinical outcomes such as survival, treatment complications, time to return to normal daily living, sustainability of health recovery, and short/long term consequences of therapies for several sets of clinical indicator conditions such as heart failure [5]. In addition to clinical outcomes, it is also important to address the concerns of the patients and their care takers. Discussions about clinical outcomes and the patients’ concerns can help understand whether the patients are likely to adopt and adhere to the clinical interventions over a long period of time.

In this volume, Ismail et al. [6] report the result of a systematic review of the effectiveness of patient-centered education in dyslipidemia management. They argued that the use of patient-centered care (PCE) is beneficial and superior to the usual form of care in dyslipidemia management. They reported not only the clinical outcomes such as the cholesterol and other levels, but also others, including psychosocial/cognitive, behavioral, and other cardio-metabolic parameters [6].

Dyslipidemia is a common condition among the elderly and is linked to cardiovascular diseases, which remain the top cause of morbidity and mortality worldwide [7]. There are many interventions for dealing with dyslipidemia [8]. Statin therapy is recommended in patients with elevated levels of low-density lipoprotein cholesterol (LDL-C). Patients with an elevated LDL-C should be counseled regarding the benefits of exercise and a healthy diet. Some patients may not tolerate statins and may need other lipid lowering drugs such as PCSK9 inhibitor or other alternative therapies [7]. All of these strategies have implications in terms of cost and outcomes on the patient and their caregivers, which can determine long term adherence to treatment. Although a hierarchical framework of outcomes for the treatment of dyslipidemia has not yet been developed by the International Consortium for health Outcomes Measurement (ICHOM), the PCE concept, as advocated by Ismail et al. [6], is the approach in the right direction. In light of the need to facilitate the patients and their relatives to balance the cost and outcomes of treatment, the attempt to define evidence-based intervention options together with hierarchical outcomes, as well as to have these discussed with them, assumes particular importance. This approach is critical for achieving patient-centered high-value integrated care.
